# Plate Fixation versus Arthroscopic‐Assisted Plate Fixation for Isolated Medium‐Sized Fractures of the Greater Tuberosity: A Retrospective Study

**DOI:** 10.1111/os.12773

**Published:** 2020-10-18

**Authors:** Qi Sun, Wei Ge, Gen Li, Jie Zhou Wu, Guanghua Lu, Runmin Li, Zhenyu Zhao, Yaru Zhu, Youzhi Xu, Lei Wang, Ming Cai

**Affiliations:** ^1^ Department of Orthopaedics, Shanghai Tenth People's Hospital, School of Medicine Tongji University Shanghai China; ^2^ Department of Orthopaedics Yancheng City No. 1 People's Hospital, Yancheng Jiangsu Province China; ^3^ Department of Orthopedics, Shanghai Institute of Traumatology and Orthopedics, Ruijin Hospital, School of Medicine Shanghai Jiaotong University Shanghai China

**Keywords:** Arthroscopic‐assisted, Isolated fractures, Open reduction and internal fixation, Plate, The greater tuberosity

## Abstract

**Objectives:**

To compare the clinical outcomes of plate fixation and arthroscopic‐assisted plate fixation in patients with displaced isolated medium‐sized fractures of the greater tuberosity.

**Methods:**

From July 2013 to October 2017, patients with displaced isolated medium‐sized fractures of the greater tuberosity who underwent arthroscopic‐assisted plate fixation (ASPF group) or open reduction and internal plate fixation (ORIF group) were retrospectively reviewed and analyzed. There were 19 patients in the ASPF group and 27 patients in the ORIF group, with comparable demographic characteristics. The average age of patients was 49.4 ± 12.1 years in the ASPF group and 46.9 ± 11.4 years in the ORIF group. The shoulder function reflected by the Constant–Murley (CS) scores, the American Shoulder and Elbow Surgeons (ASES) scores, and the range of motion (ROM) in the both groups at the last follow‐up were analyzed in the study. Surgery time, postoperative pain, and postoperative complications were also reviewed.

**Results:**

A total of 46 eligible patients were included in this study. The mean follow‐up was similar for the ASPF (19.4 ± 3.7 months) and the ORIF (18.2 ± 3.2 months) groups (*P* = 0.372). All patients had achieved primary incision healing in both groups at the last follow‐up. The surgery time was 96.8 ± 11.7 min and 64.2 ± 8.3 min in the ASPF group and the ORIF group, respectively (*P* < 0.01). All the CS scores (*P* = 0.278), ASES scores (*P* = 0.426), and ROM were slightly better in the ASPF group than in the ORIF group, but they did not attain significant differences. In addition, there was no significant difference in the postoperative complication rate between the ASPF group (10.5%) and the ORIF group (18.5%) (*P* = 0.522). In the ASPF group, there was only one patient with postoperative shoulder stiffness and one case of fracture malunion. In the ORIF group, there were two cases of postoperative shoulder stiffness, two cases of fracture malunoin, and one case of subacromial impingement. Other major postoperative complications, such as fracture nonunion, pullout of the suture anchor, and screw penetration, were not observed in either group.

**Conclusion:**

Arthroscopic‐assisted plate fixation is effective and may be an alternative in the treatment of displaced isolated medium‐sized fractures of the greater tuberosity.

## Graphical abstract

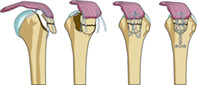

## Introduction

Proximal humeral fractures are the third most common fractures in adults^1^, ^2^, of which approximately 20% are isolated fractures of the greater tuberosity^3^. The incidence of isolated fractures of the greater tuberosity is expected to increase, because of the active lifestyles people have nowadays^4^. In fractures of the greater tuberosity, the supraspinatus and infraspinatus can induce superior displacement of the greater tuberosity, resulting in potential impingement^5^. In addition, fractures of the greater tuberosity characteristically lead to a longitudinal tear in the cuff between the supraspinatus and subscapularis tendons. The biomechanics of the rotator cuff and, subsequently, shoulder function are dramatically altered if displacement of the greater tuberosity exceeds 5 mm, even 3 mm for a population with high demands on shoulder function^6^. Fracture‐related pain and impaired range of motion (ROM) occur with the displaced fracture of the greater tuberosity after conservative treatment^7^, despite satisfactory shoulder function being achieved with minimal displaced of the greater tuberosity with conservative treatment^6^, ^8^.

Concomitant soft tissue pathologies, including rotator cuff tears, labral tears (Bankart or superior labral anterior posterior lesions), and long head of the biceps pathologies, may coexist with greater tuberosity fractures^9^. These pathologies may be overlooked otherwise, but they are easily detected and treated by arthroscopy^9^. It is essential to restore and maintain the functional reduction of the greater tuberosity for displaced fractures and to repair impaired rotator cuffs through operative management^3^. The determination of methods for treating isolated displaced fractures of the greater tuberosity largely depends on the fracture type, the bone quality, the fragment size, and the preference of the surgeon. Multiple surgical methods have been developed and described for the treatment of isolated displaced fractures of the greater tuberosity^7^, ^10^, ^11^, ^12^, ^13^, but the optimal surgical treatment remains controversial. The bony healing of the displaced greater tuberosity critically depends on anatomical reduction and rigid fixation, and the mechanical stability further allows early rehabilitation of the shoulder to achieve satisfactory clinical outcomes^8^, ^14^.

In general, open reduction and internal plate fixation (ORIF) is predominantly adopted for large‐sized isolated fractures of the greater tuberosity, especially for comminuted fractures. Although several studies show that plate fixation achieves satisfactory clinical outcomes in the treatment of isolated displaced fractures of the greater tuberosity, it is commonly associated with a high risk of complications, such as loss of reduction, especially for non‐large‐sized or comminuted fractures^15^. Despite suture bridge fixation being used in the treatment of large displaced greater tuberosity fractures^16^, arthroscopic suture bridge repair, such as double row suture anchor repair, is generally used for small‐sized fractures of the greater tuberosity^17^, ^18^, ^19^. This may be largely due to insufficient mechanical stability of suture bridge fixation for non‐small‐sized or comminuted fractures of the greater tuberosity^19^.

For comminuted or medium‐sized fractures of the greater tuberosity, the combination of suture bridge fixation and plate fixation is an alternative treatment option. Arthroscopic‐assisted plate fixation (ASPF) is described for displaced large‐sized comminuted greater tuberosity fractures in a case series^20^. There is a lack of research comparing ASPF and plate fixation or suture bridge fixation in the treatment of medium‐sized or comminuted fractures of the greater tuberosity. The present study aimed: (i) to evaluate the effectiveness of ASPF in patients with displaced isolated medium‐sized fractures of the greater tuberosity; (ii) to compare the functional outcomes of ASPF versus ORIF; (iii) to assess the rate of complications between ASPF versus ORIF in the treatment of displaced isolated medium‐sized fractures of the greater tuberosity.

## Methods and Materials

### *Design*

This is a retrospective study and was approved by our ethical review boards. Patient records and information were anonymized and de‐identified prior to analysis.

### *Time and Place*

This study was conducted from July 2013 to October 2017 at the Department of Orthopedics of Shanghai Tenth People's Hospital, School of Medicine, Tongji University.

### *Inclusion Criteria*

Inclusion criteria were as follows: (i) patients with displacement of the greater tuberosity fractures more than 5 mm but less than 2 cm; (ii) receiving plate fixation or ASPF; (iii) surgery time, American Shoulder and Elbow Surgeons (ASES) score, CS score, and complication rate were reviewed and compared; (iv) follow‐up more than 12 months; and (v) patients who provided informed consent for the treatment and the testing program.

### *Exclusion Criteria*

Exclusion criteria were as follows: concomitant injuries such as long head of biceps tendon injury or bony Bankart lesions or superior labrum anterior and posterior injury.

### *Surgical Procedure*

#### *Arthroscopic‐Assisted Plate Fixation Group*

##### Anesthesia and Position

Patients were placed in the lateral decubitus position with the traction of the injured arms under general anesthesia.

##### Arthroscopic Debridement and Repair

Joint debridement, anchor insertion, trans‐tendon repair of the rotator cuff, fracture reduction, and restoration of the medial footprint of the greater tuberosity were performed through arthroscopy, as described by Li *et al* ^21^ and Park *et al*.^20^. Debridement of the articular side of the fracture and examination for rotator cuff tears were performed before attempted reduction of fractures. If a rotator cuff tear existed, we repaired it arthroscopically.

##### Anchor Insertion

**Fig. 1:**
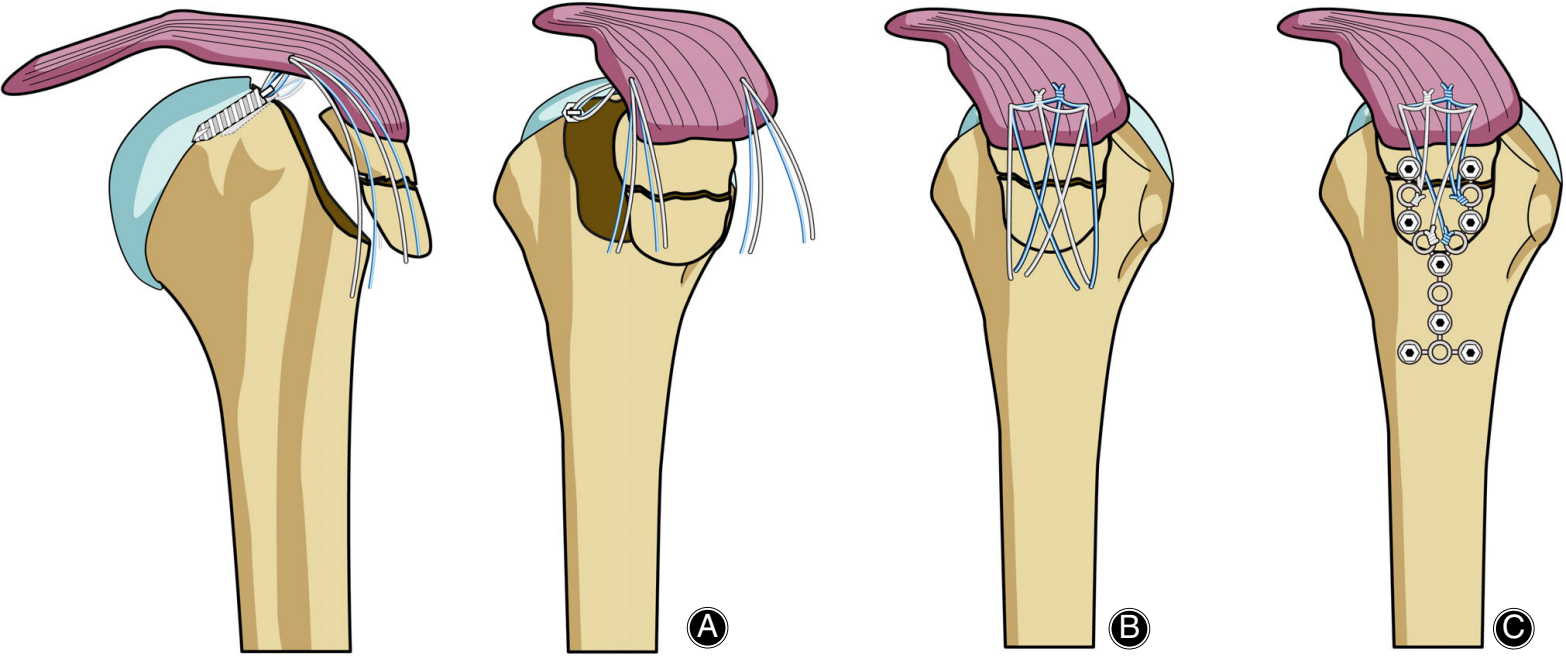
Schematic illustration of the anchor insertion, suture tightening, and plate fixation. (A) Anchor insertion through rotator cuff. (B) Two knots between the two anchors and one uncut suture strand from each knot. (C) Plate fixation and suture tightening.

After reduction, two suture anchors (TwinFix Ti; Smith & Nephew Endoscopy, Andover, MA, USA) were placed through the rotator cuff attached to the fragments and plunged into the articular edge of the humeral head (Fig. 1A). Medial row tightening was performed after the arthroscope was inserted into the subacromial space (Fig. 1B).

##### Confirmation of Restoration

Accuracy of the restoration of the medial footprint of the greater tuberosity was evaluated again after the arthroscope was inserted into the glenohumeral joint.

##### Approach and Exposure

After the arthroscopic procedure, the arm retraction was removed. A 4–6‐cm deltoid‐splitting approach was performed for further correction of the displaced greater tuberosity.

##### Reduction and Fixation

**Fig. 2:**
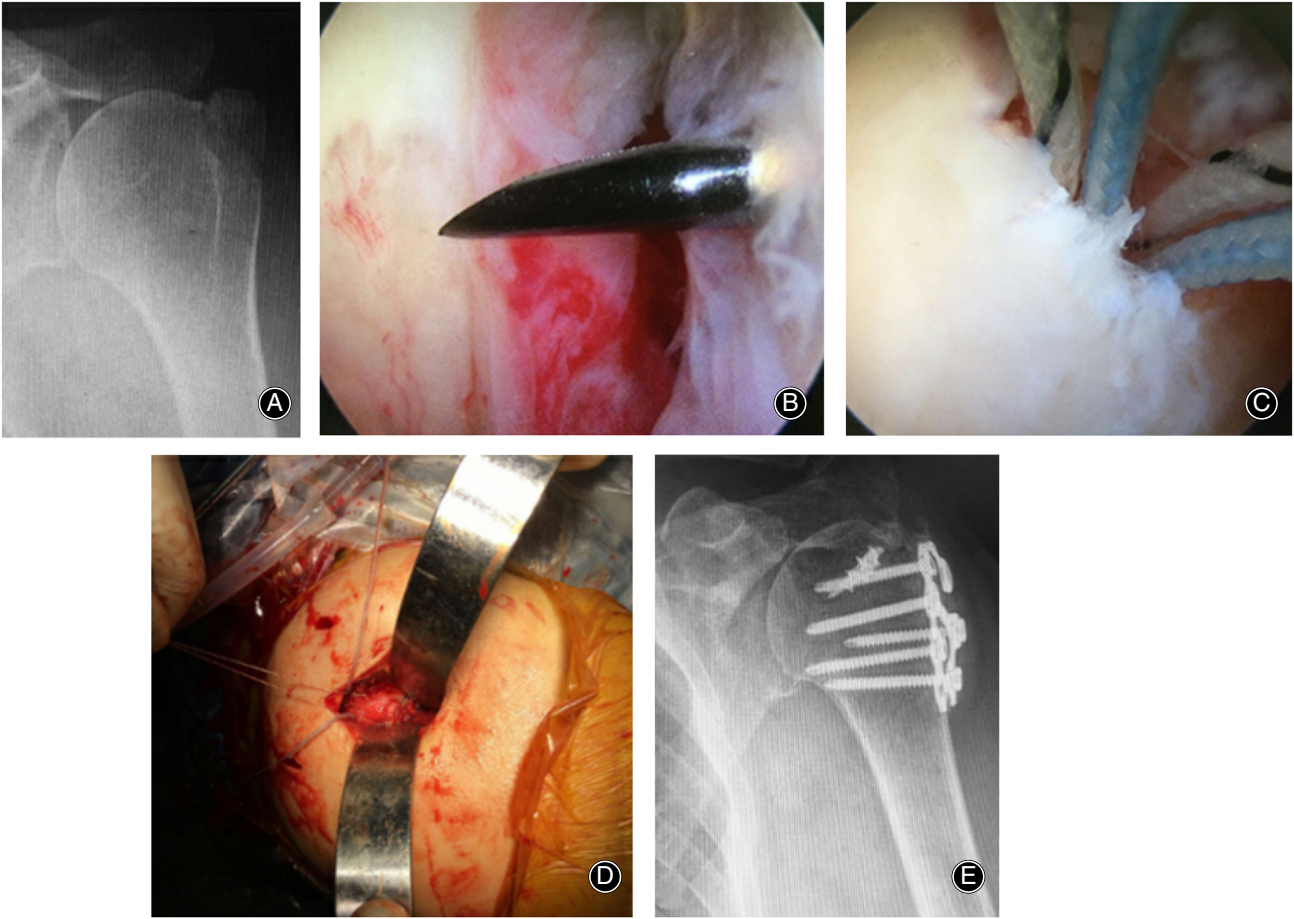
Arthroscopic‐assisted plate fixation of the isolated displaced fracture of the greater tuberosity of the left shoulder. (A) Preoperative X‐ray image. (B and C) Arthroscopic‐assisted technique. (D) Plate fixation. (E) Postoperative X‐ray image at 1‐year follow‐up.

The fracture displacement was corrected by pulling suture threads of the inserted suture anchors. A self‐adjusted calcaneus titanium plate (Litos, Hamburg, Germany) was manually bent and modulated to fit smoothly to the greater tuberosity of the proximal humerus. Subsequently, the plate was placed onto the greater tuberosity and fixed with variable screws (Fig. 1C). Cortical and cancellous screws were used as required by the surgeon. Finally, sutures from the suture anchor were tied to available holes of the plate (Fig. 2).

#### *Open Reduction and Plate fixation Group*

##### Anesthesia and Position

The patients were placed in a beach chair position under general anesthesia.

##### Approach and Exposure

A delto‐pectoral approach was performed to expose the fragments of the greater tuberosity after clearance of hematoma and scar tissue.

##### Fracture Reduction

The greater tuberosity fragments were reduced with the assistance of a heavy braided suture, which was placed into the rotator cuff. Then the proximal humeral locking plate (DePuy Synthes, Pennsylvania, USA) was positioned lateral to the bicipital groove and right beneath the tip of the greater tuberosity.

##### Fixation

**Fig. 3:**
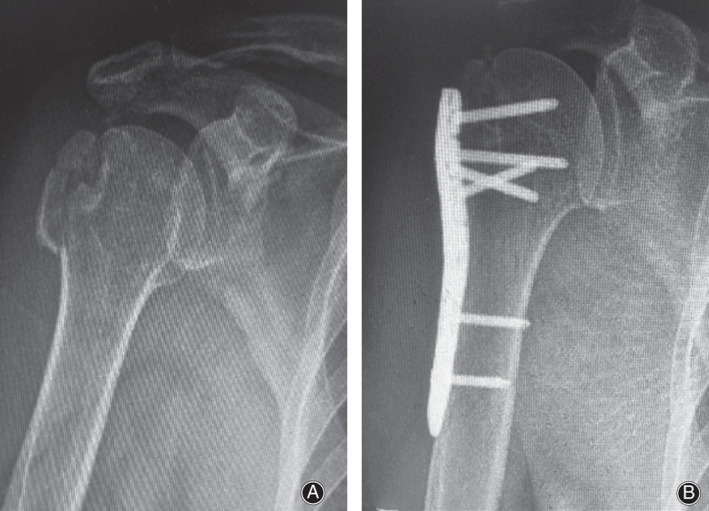
Open reduction and plate fixation of the isolated displaced fracture of the greater tuberosity of the right shoulder. (A) Preoperative X‐ray image and (B) postoperative X‐ray image at 1‐year follow‐up.

After the plate was fixed with K‐wires temporarily, intraoperative radiographs were used to confirm satisfactory reduction of the greater tuberosity and the plate position. Subsequently, proximal locking screws were inserted unicoritcally and distal locking screws were inserted bicortically, which were confirmed by intraoperative radiographs. Finally, braided sutures were tied to the plate through the suture eyelets for supplemental fixation of the greater tuberosity fracture (Fig. 3).

### *Postoperative Management*

The postoperative rehabilitation regime was similar for the ASPF and ORIF groups. The affected upper extremity was supported by a sling bandage for 4 weeks after surgery. The patients were allowed to begin passive exercises from the second postoperative day. The active assistant exercises were initiated from 6 weeks after surgery. Active resistance exercises were usually started 3 months postoperatively or when radiographic evidence showed fracture healing.

### *Follow‐up and Data Collection*

#### *Intraoperative Evaluation*

Surgery time was recorded in this study, which was defined as the time from initiation to closure of the incision. Surgery time is one index to assess the difficulty of surgery.

#### *Radiological Evaluation*

Conventional antero–posterior and lateral radiographs of the shoulder of the injured side were taken to assess radiological outcomes at every follow‐up. The radiographs were used to evaluate the union, malunion, non‐union, or status of fracture reduction.

#### *Clinical Evaluation*

##### Visual Analog Scale

The visual analog scale (VAS) is the most commonly used questionnaire for quantification of pain. It is a continuous scale comprised of a horizontal or vertical line, usually 10 cm in length. For pain intensity, the scale is most commonly anchored by “no pain” (score of 0) and “pain as bad as it could be” (score of 10). A score of 0 is considered no pain, 1–3 mild pain, 4–6 moderate pain, and 7–10 severe pain.

##### Constant–Murley score

The Constant–Murley (CS) score is recommended by the executive committee of the European Society for Surgery of the Shoulder and the Elbow as an assessment method to evaluate overall shoulder function. In detail, the CS scale is a comprehensive tool for evaluation of shoulder function based on four aspects related to shoulder pathology: two subjective, pain and activities of daily living; and two objective, range of motion and strength.

##### American Shoulder and Elbow Surgeons Score

The ASES score is another useful mixed outcome reporting measure for all patients with shoulder pathology. The psychometric properties of the ASES have been well established and its validity, reliability, and responsiveness have been assessed in a variety of shoulder problems, including: rotator cuff disease, glenohumeral arthritis, shoulder instability, and shoulder arthroplasty.

##### Range of Motion

The ROM, including forward flexion, abduction, external rotation, and internal rotation, at the last follow‐up were reviewed. Using the individual value of the ROM is more straightforward for showing the functional outcome of the injured shoulder.

##### Occurrence of Complications

Complications, including technique‐related and non‐related compilations, in each group were also analyzed during the follow‐up period.

### *Statistical Analysis*

The SPSS 18.0 software package (IBM, Chicago, USA) was used for statistical analysis in this study. All continuous variables (age, interval from injury to surgery, follow‐up, surgery time, CS score, ASES score, VAS, forward flexion, abduction, and external rotation) were expressed as mean ± standard deviation, categorical variables (gender and fracture status) were expressed as frequency (percentage), and internal rotation was expressed as median. The χ^2^‐test was used to compare categorical variables and the Student *t*‐test was used to compare continuous variables between the ASPF group and the ORIF group. *P* < 0.05 was regarded as statistically significant.

## Results

### *Patient Characteristics*

A total of 46 eligible patients were included in this retrospective study. There were 19 patients with an average age of 49.4 ± 12.1 years in the ASPF group and 27 patients with an average age of 46.9 ± 11.4 years in the ORIF group (*P* = 0.471). The male to female ratio of the two groups was also similar: there were 13 men and six women in the ASPF group, and 20 men and seven women in the ORIF group (*P* = 0.675) (Table 1). The causes of injury included traffic accident injury (21), sport injury (10) and falling injury (15), which were comparable between the ASPF group and the ORIF group. Regarding fracture status of the greater tuberosity, there were 15 patients with non‐comminuted fractures of the greater tuberosity and four with comminuted fractures in the ASPF group; there were 21 patients with non‐comminuted fractures of the greater tuberosity and six with comminuted fractures in the ORIF group, with no significant difference (*P* = 0.925) (Table 1). The mean interval from injury to surgery was 7.9 ± 4.4 days in the ASPF group and 8.2 ± 4.3 days in the ORIF group (*P* = 0.498). The mean follow‐up was similar for the ASPF group and the ORIF group: 19.4 ± 3.7 months for the ASPF group and 18.2 ± 3.2 months for the ORIF group (*P* = 0.372).

**TABLE 1:** Baseline characteristics of patients with displaced, isolated medium‐sized fractures of the greater tuberosity

Variables	ASPF group (n = 19)	ORIF group (n = 27)	*P*‐value
Gender			0.675
Male	13	20	‐
Female	6	7	‐
Age (years)	49.4 ± 12.1	46.9 ± 11.4	0.471
Fracture status			0.925
Non‐comminuted fractures	15	21	‐
Comminuted fractures	4	6	‐
Interval from injury to surgery (days)	7.9 ± 4.4	8.2 ± 4.3	0.498
Follow‐up (months)	19.4 ± 3.7	18.2 ± 3.2	0.372

ASPF, arthroscopic‐assisted plate fixation; ORIF, open reduction and internal fixation.

Gender and fracture status were expressed as frequency (percentage). Age, interval from injury to surgery, and follow‐up are expressed as means ± standard deviation.

### *Functional Outcomes*

All patients had achieved primary incision healing in both groups at the last follow‐up.

#### *Surgery Time*

Compared with the ORIF group, the surgery time was significantly longer in the ASPF group (96.8 ± 11.7 min in the ASPF group and 64.2 ± 8.3 min in the ORIF group, *P* < 0.01).

#### *Constant–Murley and The American Shoulder and Elbow Surgeons Score*

The shoulder function was evaluated using the CS and ASES scores. Although there was lack of significant differencein CS scores (*P* = 0.278) and in ASES scores (*P* = 0.426) between the ASPF group and the ORIF group, the CS scores and ASES scores were slightly higher in the ASPF group than in the ORIF group (Table 2.). The CS score (86.2 ± 7.9) and the ASES score (84.5 ± 8.9) in the ASPF group were higher than those (CS: 80.9 ± 7.5, ASES: 79.1 ± 8.1) in the ORIF group by 6.6% and 6.8%, respectively.

**TABLE 2:** Clinical outcomes of arthroscopic‐assisted plate fixation (ASPF group) and plate fixation (ORIF group) for displaced isolated medium‐sized fractures of the greater tuberosity

		ASPF group	ORIF group	*P‐*value
Surgery time (min)		96.8 ± 11.7	64.2 ± 8.3	<0.01
CS score		86.2 ± 7.9	80.9 ± 7.5	0.278
ASES score		84.5 ± 8.9	79.1 ± 8.1	0.426
VAS		0.8 ± 0.7	0.9 ± 0.9	0.334
ROM (°)				
Forward flexion		152.2 ± 14.5	148.9 ± 11.6	0.649
Abduction		144.2 ± 16.7	135.9 ± 18.8	0.058
External rotation		41.4 ± 6.4	38.2 ± 6.2	0.053
Internal rotation		L_1_	L_2_	0.475

ASES score, American Shoulder and Elbow Surgeons score; CS score, Constant‐Murley score; L, level of lumbar spine; surgery time; ROM, range of motion; VAS, visual analog scale. CS score, ASES score, VAS, forward flexion, abduction, and external rotation were expressed as mean ± standard deviation. Internal rotation was expressed as the class mid‐value.

#### *Range of Motion*

For ROM, all indices, including forward flexion (*P* = 0.649), abduction (*P* = 0.058), external rotation (*P* = 0.053), and internal rotation (*P* = 0.475), were also better in the ASPF group than the ORIF group, despite non‐significant difference. In particular, the abduction and external rotation of the affected shoulder were 144.2 ± 16.7 and 41.4 ± 6.4 in the ASPF group and 135.9 ± 18.8 and 38.2 ± 6.2 in the ORIF group. Compared with the ORIF group, abduction and external rotation increased more in the ASPF group, by 6.1% and 8.4%, respectively.

#### *Visual Analog Scale* *Score*

In addition, the VAS scores were 0.8 ± 0.7 and 0.9 ± 0.9 in the ASPF and ORIG groups, respectively (*P* = 0.475). Although these data suggested that the postoperative pain was similar between these two groups, the VAS revealed 12.5% improvement in the ASPF group.

### *Complications*

We did not observe significant differences in complication rates between the ASPF group and the ORIF group. In the ASPF group, there was only one patient with postoperative shoulder stiffness and one case of fracture malunion. In the ORIF group, there were two case of postoperative shoulder stiffness, two cases of fracture malunion, and one of subacromial impingement. To improve shoulder function, the three patients with postoperative shoulder stiffness were manipulated to release adhesion under anesthesia. All three patient obtained satisfactory shoulder function after manipulation, based on their self‐evaluation. The patient with subacromial impingement had subsequent relief of impingement symptoms after removal of the plate. The patient with fracture malunion in the ASPF group refused to undergo reoperation. The two patients with fracture malunion in the ORIF group underwent reoperation, and stable fixation and bone union was achieved. In the current study, other major postoperative complications, such as fracture nonunion, pullout of the suture anchor, and screw penetration, were not observed in either group.

## Discussion

Non‐displaced isolated fractures of the greater tuberosity can be successfully treated by conservative treatment^3^. For displaced isolated fractures of the greater tuberosity, surgical treatment can contribute to better shoulder function, higher patient satisfaction, and lower rates of reoperation and complications^22^, ^23^, ^24^. Even though various surgical techniques are available, the optimal choice for the treatment of displaced isolated medium‐sized fractures of the greater tuberosity has not been determined. Our study demonstrated that both ASPF and ORIF achieved satisfactory reduction of the greater tuberosity and provided relatively stable fixation. Although the CS scores, ASES scores, and ROM were comparable between the ASPF group and the ORIF group, they were slightly better in the ASPF group. Considering the small sample and differences in the study, the results should be interpreted cautiously. Moreover, the complication rate is similar for the ASPF group and the ORIF group.

### *Functional Outcome*

A favorable clinical outcome of plate fixation was reported for displaced isolated fractures of the greater tuberosity^3^, ^7^. Arthroscopic double‐row suture anchor fixation was also reported to be effective in the treatment of greater tuberosity fractures, because of a biomechanical advantage and accurate trans‐tendon repair of the rotator cuff^25^, ^26^, ^27^. However, the size of greater tuberosity fragments differ; the plate is mainly used for large‐sized fractures and the arthroscopic fixation is largely used for relatively smaller sized fractures. A previous study compared the arthroscopic technique and plate fixation in the treatment of displaced isolated fractures of the greater tuberosity; it reported that there were few important differences between the two fixation techniques^19^ Park *et al*.^20^. reported a similar arthroscopic‐assisted plate fixation technique for displaced isolated fractures of the greater tuberosity, with a mean ASES score of 83.8, which is comparable to our result in the ASPF group (mean ASES: 84.5). Medium‐sized fractures where fracture lines do not exceed the surgical neck are investigated in the study. Compared with the arthroscopic double‐row suture anchor, plate fixation could provide rigid fixation and cover a larger fracture area^5^, ^28^. As demonstrated in our study, the CS scores, the ASES scores, and the ROM in the ASPF group were comparable to the corresponding variables in the ORIF group. Therefore, both plate fixation and arthroscopic‐assisted plate fixation were effective treatments for displaced isolate medium‐sized fractures of the greater tuberosity.

The CS scores, the ASES scores, and the ROM were slightly better in the ASPF group. Considering the slight differences and potential bias in the study, the results should be interpreted cautiously. ASPF is more likely to be recommended for the treatment of displaced isolated medium‐sized fractures of greater tuberosity in young patients or active patients who have a higher requirement for shoulder function. The potential advantages with ASPF in the treatment of displaced isolated medium‐sized fractures of the greater tuberosity may partially explain the slightly better shoulder function. First, the arthroscopic‐assisted fixation with a double‐row suture anchor was more effective in reduction of displacement of the greater tuberosity and restoration of the medial footprint of the greater tuberosity; the large displacement of the greater tuberosity limited ROM^20^, ^29^. Second, the suture anchor of arthroscopic‐assisted plate fixation provides a supplemental fixation to resist this traction force of the infraspinatus and the teres minor muscles^29^. Compared with plate fixation, the ASPF allows early rehabilitation.

In several previous studies, arthroscopic double‐row suture anchor fixation was associated with a longer surgery time and learning curve^12^, ^30^. The hybrid technique of arthroscopic double‐row suture anchor fixation and plate fixation in our study also had a longer surgery time than plate fixation. We believe longer surgery time might be correlated with higher potential risk of surgery. In addition, ASPF needs orthopaedic surgeons to be familiar with both arthroscopic techniques and ORIF, suggesting that it is more technically demanding.

### *Analysis of Complications*

The postoperative complication rate was comparable between the ASPF group and the ORIF group. Liao *et al*. (2016) compared the arthroscopic double‐row suture anchor fixation with locking plate fixation in patients with isolated displaced fractures of the greater tuberosity; they also reported similar differences in complication rates between them^19^. Subacromial impingement was a common complication when the locking plate was used in the treatment of isolated displaced fractures of the greater tuberosity^31^. For fractures of the greater tuberosity with a small or medium sized fragment, the locking plate is usually placed in a relatively proximal position to ensure firm fixation of fractures of the greater tuberosity. However, that might increase the incidence of secondary subacromial impingement. The ASPF provides additional fixation by suture bridge, which may avoid potential subacromial impingement by allowing a lower position of the lateral plate. Although subacromial impingement was not observed in the ASPF group, attention should also be paid to the position of the calcaneus titanium plate. Fracture malunion occurred in one patient in the ASPF group and two patients in the ORIF group. All of them occurred with comminuted fractures of the greater tuberosity, which is largely caused by insufficient stability. There was one case of postoperative shoulder stiffness in the ASPF group and two cases in the ORIF group, which might partially be because of the potential poor compliance with rehabilitation. Furthermore, a more active rehabilitation program with early mobilization may be required if the stability of fixation allows it.

### *Limitations of the Study*

This study had several limitations. First, this study was a retrospective study, so there is potential selection bias. Second, we did not conduct an analysis of the cost effectiveness of the two fixation groups, because the price of implants was not accessible for clinical surgeons. Third, arthroscopic‐assisted plate fixation is not a standard fixation treatment for displaced isolated fractures of the greater tuberosity, and is highly technically demanding. Fourth, the study only included medium‐sized fractures of the greater tuberosity, but the exact size varied, thus inducing potential bias. Furthermore, the approaches were the deltoid‐splitting and the delto‐pectoral approach in the ASPF group and the ORIF group, respectively, which might induce potential bias of clinical efficacy. However, given the nature of retrospective studies, the bias cannot be avoided. Finally, although all parameters of shoulder function are better with arthroscopic‐assisted plate fixation, this result may partially due to the small sample size in this study. A long‐term prospective randomized controlled trial with a large sample is needed to clarify this issue for identification of potential differences.

### *Conclusion*

Both plate fixation and arthroscopic‐assisted plate fixation were effective in the treatment of displaced isolated medium‐sized fractures of the greater tuberosity. Although arthroscopic‐assisted plate fixation is technically demanding, it may be an alternative for isolated medium‐sized fractures of the greater tuberosity with satisfactory clinical outcomes.
